# Rush Medical College

**Published:** 1857-10

**Authors:** 


*Rush Medical College.*

The prospects of a good class in this institution are fully equal to those of any former year.

The preliminary course for the present month commenced on Tuesday the 6th inst., and consists of clinical instruction in the Mercy Hospital every morning; clinics, in the College by Professors Brainard and Byford, on Wednesdays and Saturdays; and two lectures per day in the College during the remaining four days of the week. The Lectures embrace the following topics, viz; Histology, by Prof. Freer; Pathology of the Urine, by Prof. Johnson; Diseases of the Os and Convex Uteri, by Prof. Byford; and Physical Diagnosis, by Prof. Davis. If we add to these lectures and clinics abundant facilities for dissection, it will be apparent that our city offers advantages to the medical student fully equal to those found elsewhere during the present month. Nor are these advantages unappreciated.

For, though but one week has elapsed since the commencement of the course, already the number of students in attendance is double that which attended the much advertised Summer Course of Clinical instruction in Detroit; and which was to mark "an era" in medical teaching; or, according to Dr. Pitcher, constitute the *first step* towards bringing back the Clinical instruction in America, to the Hippocratic method.

